# Editorial: Neuroscience Horizons: Perspectives from AfrAbia

**DOI:** 10.1016/j.ibneur.2026.09.020

**Published:** 2026-09-28

**Authors:** Wael Mohamed

**Affiliations:** a Basic Medical Science Department (BMS), Kulliyyah of Medicine (KOM), International Islamic University Malaysia (IIUM), Pahang, Malaysia; b Clinical Pharmacology Department, Menoufia Medical School, Menoufia University, Menoufia, Egypt

Neuroscience research within the AfrAbia regions is undergoing a dynamic evolution, transitioning from an underrepresented field into a vital contributor to global neurological and psychiatric sciences. Researchers across these nations are actively addressing diverse neurobiological challenges, ranging from neurodegeneration to computational neuroscience. This special issue, **Neuroscience Horizons: Perspectives from AfrAbia**, is of critical importance; it not only spotlights the rigorous, innovative research emerging from these regions but also helps bridge the geographical gap in global scientific literature, ensuring that regionally led insights reach the broader international community.

This multidisciplinary issue highlights the breadth of neuroscience research across the AfrAbia region, encompassing molecular pathology, neuropharmacology, clinical interventions, and computational methodologies. Contributions advancing the understanding of neurodegeneration and brain aging include the identification of novel cellular markers for Alzheimer's disease (Alobu et al.), explorations of alternative splicing mechanisms in dementia (Aremu), and analyses of CLN3 disease pathology (Bilal et al.). The issue prominently features neuropharmacological research, detailing the anti-inflammatory and neuroprotective efficacies of *Saccharomyces boulardii* (Akhbari et al.), taurochenodeoxycholic acid (Wang et al.), and *Moringa oleifera L.* (Alhawiti et al.), alongside findings on CB2 receptor modulation in morphine tolerance (Omar et al.). Clinical and psychiatric sciences are represented through machine learning applications for depression classification (Salimi et al.), meta-analyses linking Vitamin D to brain volumetrics (Tamannaeifar et al.) and restless legs syndrome to inflammatory bowel disease (Banihashemian et al.), as well as therapeutic evaluations for multiple sclerosis (Savaedi et al., Aghdam et al.). Finally, investigations into the neurobiological mechanisms of sleep deprivation on memory (Adeniji et al.) and the capacity for computational neuroscience in Nigeria (Alex et al.) underscore the region's expanding methodological diversity and scientific impact

This special issue significantly advances the development of neuroscience in AfrAbia by demonstrating the region's capability to produce high-impact, multidisciplinary research spanning neuropharmacology, molecular biology, and computational neuroscience. Despite these advancements, neuroscience research across the AfrAbia region faces significant systemic challenges, notably severe funding constraints, inadequate laboratory infrastructure, limited access to advanced technologies, and the ongoing brain drain of skilled researchers. To overcome these barriers and accelerate scientific progress, key interventions must focus on establishing sustainable regional funding mechanisms, developing shared high-tech core facilities, and fostering equitable international research partnerships. Furthermore, expanding capacity-building programs, improving access to open-access publishing, and incentivizing talent retention will be crucial to strengthening local expertise and elevating the global visibility of AfrAbian neuroscience. To further increase the visibility and impact of AfrAbian neuroscience, future efforts must prioritize establishing sustained international research partnerships, expanding grant opportunities specifically for researchers in these regions, and investing heavily in local computational and laboratory infrastructure. Additionally, supporting open-access publication models will be vital to ensuring that discoveries from AfrAbia continue to inform and enrich the global neuroscience community.

